# In this month’s Bulletin

**DOI:** 10.2471/BLT.24.000524

**Published:** 2024-05-01

In the editorial section, Petteri Orpo and Tedros Adhanom Ghebreyesus (298) introduce the work of WHO’s Council on the Economy of Health for All, the subject of this special theme issue. Ritu Sadana et al. (299) summarize the contents of the theme issue and explain (300) how the council reframes economic goals in pursuit of health.

Gary Humphreys (303–304) reports on the financing implications of protracted humanitarian responses and interviews Derrick Sim (305–306) about vaccine supply and demand during the COVID-19 pandemic. 

## Argentina, Australia, Brazil, Canada, China, European Union, France, Germany, India, Indonesia, Italy, Japan, Mexico, Republic of Korea, Russian Federation, Saudi Arabia, South Africa, Türkiye, United Kingdom, United States of America

### Countering pandemic threats 

Raymond Hutubessy et al. (366–367) describe collaboration between finance and health ministers.

## Argentina, Bangladesh, Brazil, Egypt, India, Indonesia, Kenya, Nigeria, Pakistan, Senegal, Serbia, South Africa, Tunisia, Ukraine, Viet Nam

### Reaching economic sustainability 

Devika Dutt et al. (344–351) present an mRNA technology transfer programme.

## Brazil 

### Universal access to health care 

Carlos AG Gadelha et al. (352–356) make the case for facilitating production and innovation.

## GLOBAL

### Tracking the right to health

Alicia Ely Yamin et al. (307–313) analyse governments’ progress.

### Who pays for pandemic preparedness?

Roberto Duran-Fernandez et al. (314–322) review the evidence on the financing of response measures.

### Coordinating health, social and economic policies 

Jo-An Occhipinti et al. (323–329) provide a blueprint for national mental wealth observatories.

### Understanding climate finance and health

Josephine Borghi et al. (330–335) explore multilateral funds, voluntary market-based mechanisms, taxes, microlevies and adaptive social protection. 

### Breastfeeding to reduce carbon emissions

Julie Patricia Smith et al. (336–343) propose that relevant investments count towards countries’ carbon offsets.

### Growth in human and environmental terms

Mark Hanson et al. (357–359) consider revised measures of economic activity.

### Creating a health-financing taxonomy

Vanessa Huang et al. (360–362) call for clarity on priorities and nomenclature.

### Health as a function of climate mitigation 

Maarten Oranje and Inke Mathauer (363–365) outline a financing policy agenda.

### Building resilient health systems 

Chris James et al. (368–369) argue for structured dialogue between health and finance sectors.

### Paying for action on antimicrobial resistance 

Serife Genc Ileri et al. (370–372) investigate financing options for national plans.

